# Draft Genome Sequence of Lactobacillus mulieris UMB7784, Isolated from the Female Urinary Tract

**DOI:** 10.1128/MRA.00403-20

**Published:** 2020-05-21

**Authors:** Oleksandra Tsibere, Taylor Miller-Ensminger, Adelina Voukadinova, Alan J. Wolfe, Catherine Putonti

**Affiliations:** a Department of Biology, Loyola University Chicago, Chicago, Illinois, USA; b Bioinformatics Program, Loyola University Chicago, Chicago, Illinois, USA; c Department of Microbiology and Immunology, Stritch School of Medicine, Loyola University Chicago, Maywood, Illinois, USA; d Department of Computer Science, Loyola University Chicago, Chicago, Illinois, USA; Queens College

## Abstract

We sequenced the genome of *Lactobacillus mulieris* UMB7784, isolated from the urinary tract. With a genome size of 1,695,489 bp and a GC content of 34.2%, the draft genome sequence presented here expands our understanding of lactobacilli in the female bladder.

## ANNOUNCEMENT

*Lactobacillus* species are native members of the female urinary microbiota (1–3), and their presence is associated with a healthy urinary tract (4, 5). Some *Lactobacillus* species can inhibit the growth of uropathogens by releasing biosurfactants with antimicrobial properties (6, 7). One such species, Lactobacillus jensenii, releases biosurfactants that disrupt biofilms of pathogens, such as Enterobacter aerogenes and Escherichia coli (8). In March 2020, L. jensenii was split into two species—L. jensenii and *L. mulieris*—based upon average nucleotide identity (9). The type strain for the new species *L. mulieris* was isolated from a urine sample, and here, we present the genome sequence of another urinary isolate of this new lineage. *L. mulieris* UMB7784 was isolated from the catheterized urine of a woman with a recurrent urinary tract infection (UTI).

Catheterized urine samples were collected from women as part of a prior institutional review board (IRB)-approved study (University of California, San Diego, IRB no. 170077AW). *L. mulieris* UMB7784 was isolated using the expanded quantitative urinary culture (EQUC) protocol (10). The genus and species were identified by matrix-assisted laser desorption ionization–time of flight (MALDI-TOF) mass spectrometry, following the protocol previously detailed (10), prior to storage at −80°C. The isolate was streaked onto a Columbia nalidixic acid (CNA) agar plate and incubated for 24 hours at 35°C in 5% CO_2_. A single colony was transferred to liquid MRS medium supplemented with 1 ml/liter of Tween 80 and grown for 48 hours at 35°C in 5% CO_2_. DNA was extracted using the DNeasy blood and tissue kit. Some modifications were made to the manufacturer’s Gram-positive procedure, as follows: 230 μl of lysis buffer (180 μl of 20 mM Tris-Cl, 2 mM sodium EDTA, and 1.2% Triton X-100 and 50 μl of lysozyme) was used (step 2), and the sample was incubated at 56°C for 10 minutes (step 5). DNA was quantified using a Qubit fluorometer. Sequencing was performed by the Microbial Genome Sequencing Center (MiGS) at the University of Pittsburgh. DNA was first enzymatically fragmented using an Illumina tagmentation enzyme, and indices were attached using PCR. Sequencing was conducted on the Illumina NextSeq 550 platform, producing 2,368,077 pairs of 150-bp reads. Raw reads were trimmed using Sickle v1.33 (https://github.com/najoshi/sickle) and assembled using SPAdes v3.13.0 with the “only-assembler” option for k values of 55, 77, 99, and 127 (11). The coverage was calculated using BBMap v38.47 (https://sourceforge.net/projects/bbmap/). The genome was annotated using PATRIC v3.6.3 (12) and the NCBI Prokaryotic Genome Annotation Pipeline (PGAP) v4.11 (13). Unless otherwise noted, default parameters were used for all software tools.

The *L. mulieris* UMB7784 draft genome is 1,695,489 bp long in 42 contigs with a 345× genome coverage and an *N*_50_ score of 67,456 bp. The genomic GC content is 34.2%. PGAP annotation identified 1,451 protein-coding genes, 53 tRNA genes, and 5 rRNA genes. PHASTER (14) analysis revealed 1 incomplete phage, and CRISPRCasFinder v1.1.2 (15) detected 1 CRISPR array with 40 spacer sequences. Since our strain was isolated from the urine of a woman with a UTI, further analysis will be useful for studying the antimicrobial properties of *L. mulieris*. Future work will expand our understanding of the prevalence and role(s) of this new species within the female urinary tract.

### Data availability.

This whole-genome shotgun project has been deposited in GenBank under the accession no. JAAVSF000000000. The version described in this paper is the first version, JAAVSF010000000. Raw sequence data are publicly available under accession no. SRR11441026.
